# Inflammatory biomarkers refine progression risk stratification in NSCLC patients with stable disease

**DOI:** 10.1016/j.tranon.2026.102769

**Published:** 2026-04-20

**Authors:** Kleinberger M, Laengle S, Berger JM, Gottmann L, Sunder-Plassmann V, Korpan M, Solano Henao I, Fuerst J, Starzer AM, Berchtold L, Tomasich E, Preusser M, Furtner J, Berghoff AS

**Affiliations:** a Department of Medicine I, Division of Oncology, Medical University of Vienna, Austria; b Christian Doppler Laboratory for Personalized Immunotherapy, Department of Medicine I, Medical University of Vienna, Austria; c Center for Medical Statistics, Informatics and Intelligent Systems, Medical University of Vienna, Austria; d Department of Biomedical imaging and Image-guided Therapy, Division of General and Paediatric Radiology, Medical University of Vienna, Austria

**Keywords:** Radiological imaging, Lung cancer, RECIST, Stable disease, Biomarker

## Abstract

**Introduction:**

Early risk stratification of non-small cell lung cancer (NSCLC) patients with stable disease (SD) at first restaging is particularly challenging. We explored the prognostic value of clinical and inflammatory markers in this population.

**Methods and Material:**

We analysed a real-world cohort of prospectively enrolled advanced NSCLC patients undergoing systemic intravenous anticancer treatment in a palliative intent at the *Medical University of Vienna* between 2019 and 2024. Inflammatory blood markers were measured at baseline and first restaging, with blinded radiologic assessment. Uni- and multivariable logistic regression models evaluated associations with durable clinical benefit (DCB).

**Results:**

Eighty NSCLC patients with SD at first restaging were included (median age 65 years, 50% female). Of those, 41 (51.3%) achieved DCB. Baseline characteristics were largely comparable. Patients with DCB had lower baseline neutrophil-to-lymphocyte and lymphocyte-to-leukocyte ratios. At first follow-up, CRP was lower and albumin higher in patients with DCB. In univariable analysis, lower follow-up albumin and higher LDH were associated with reduced odds of DCB. In multivariable models, PD-L1 positivity and follow-up albumin remained associated with DCB. The combined clinical-inflammatory model showed the highest apparent discriminative performance (AUC 0.766), compared to clinical-only (AUC 0.657) and inflammatory-only models (AUC 0.727), although the incremental improvement was modest.

**Discussion:**

In patients with advanced NSCLC and SD at first restaging, inflammatory biomarkers were associated with additional discriminative information beyond clinical characteristics alone. A combined clinical-inflammatory model showed numerically higher discriminative performance; however, the improvement was modest.

## Highlights

• Stable disease (SD) at early imaging is common in non-small cell lung cancer.
• About half of patients with early SD later achieve durable clinical benefit (DCB).
• Lower inflammation and higher albumin at baseline and follow-up associate with DCB.
• Clinical-blood marker combinations outperform single-domain models for later DCB.

## Introduction

Non-small cell lung cancer (NSCLC) represents approximately 85% of all lung malignancies and remains a major cause of cancer-related death worldwide [1,2]. The introduction of immune checkpoint inhibitors (ICIs) has significantly improved survival for subsets of patients, however, clinical outcomes remain highly variable and only 20-40% of treated individuals achieve lasting treatment response [3,4]. Identifying biomarkers that can optimize patient selection and predict immunotherapy response is therefore an ongoing clinical priority [5].

Current treatment stratification primarily relies on PD-L1 expression and molecular profiling like tumor mutational burden (TMB) or microsatellite instability (MSI), yet these factors explain only part of the heterogeneity in treatment response [6,7]. There is growing evidence that systemic inflammation and circulating tumor markers may provide complementary information beyond tissue-based biomarkers [5]. Among these, inflammatory markers as well as ratios between immune cell counts have emerged as promising serum biomarkers for treatment response in NSCLC. These include well-established indices such as the neutrophil-to-lymphocyte ratio, as well as less commonly used parameters such as the lymphocyte-to-leukocyte ratio, which reflects the relative contribution of lymphocytes within the total leukocyte compartment and may serve as an exploratory marker of systemic immune balance. However, optimal stratification methods, particularly for patients with stable disease (SD) at early restaging, remain unclear. The current literature underscores the critical need for reliable biomarkers capable of distinguishing transient disease control from durable clinical benefit (DCB), especially in patients with early SD, a group for whom radiologic assessment alone is insufficient [8].

Beyond their biological relevance, serum markers offer practical advantages: they are routinely available, inexpensive and allow easy serial monitoring, making them attractive candidates for integration into real-world risk models [5]. Importantly, previous analyses have shown that inflammatory serum parameter, such as C-reactive protein (CRP) correlate strongly with poorer treatment outcome and shorter survival [9]. Combining clinical variables with serum-based inflammatory parameters may provide an improved characterization of the interplay between tumor biology and host inflammatory response.

Nevertheless, further research is required to develop robust approaches for subdividing patients with SD more precisely. The present exploratory study therefore investigated the potential value of clinical and inflammatory parameters in a real-world cohort of patients with advanced NSCLC, with a specific focus on those presenting with radiologic SD at first restaging. By analyzing these markers at baseline (BL) and at first follow-up/restaging (FU), we aimed to assess whether routinely available clinical and serum-based biomarkers may support early risk stratification within this clinically relevant and understudied subgroup.

## Materials and methods

### Study design

This study was performed as an exploratory, hypothesis-generating study on an institutional real-world cohort of prospectively enrolled patients treated at the *Division of Oncology, Department of Medicine I, Medical University of Vienna* between 01/2019 and 12/2024. The present study was approved by the ethics committee of the *Medical University of Vienna* (ethics committee number 2065/2022) and conducted in accordance with the Declaration of Helsinki 2008.

### Patient selection

All enrolled patients were prospectively included in the “*Translational Research Unit (TRU) Biobanking Program for Personalized Immunotherapy”* (ethics committee number 1164/2019), a prospective, observational study collecting clinical data and biological specimens. All included patients were ≥ 18 years of age, had histologically confirmed NSCLC of locally advanced or metastatic stage and received intravenous systemic anti-neoplastic treatment regimen in a palliative intend.

### Inflammatory parameters

Inflammatory laboratory parameters were obtained through systematic chart review at two predefined time points: the start of systemic therapy (= baseline, BL) and the first radiologic restaging (= follow-up, FU). For each biomarker, the absolute values at BL and FU were extracted, and both absolute and relative changes between these time points were calculated. These included neutrophil-to-lymphocyte ratio (NLR), leukocyte-to-lymphocyte ratio (LLR), platelet-to-lymphocyte ratio (PLR), monocyte-to-lymphocyte ratio (MLR) and the CRP/Albumin ratio (CAR). The LLR was included as an exploratory parameter reflecting the proportion of lymphocytes within the total leukocyte count, conceptually related to other lymphocyte-based indices of systemic immune balance. In addition, biochemical inflammatory markers comprising CRP, Albumin and lactate dehydrogenase (LDH) were recorded. Absolute change was defined as the difference between FU and BL values, whereas relative change was calculated as the percentage difference from baseline.

### Radiological response evaluation

Treatment response was assessed blinded to clinical outcomes by a board-certified radiologist. Restaging of patients treated with immune checkpoint inhibitors (monotherapy or in combination with chemotherapy and/or targeted therapy) was evaluated according to the iRECIST guideline [10], while patients who did not receive ICIs were assessed in line with RECIST v1.1 criteria [11]. DCB was defined as sustained disease control ((i)CR, (i)PR or (i)SD) lasting ≥ 6 months after treatment initiation. This endpoint is conditional on patients remaining progression-free and alive long enough to meet this definition and was therefore used to discriminate outcomes within the subgroup of patients with stable disease at first restaging rather than to model long-term survival.

### Statistical analysis

Descriptive statistics were used to summarize patient characteristics, treatment information and biomarker values. Continuous variables were reported as medians with ranges and categorical variables as absolute frequencies and percentages. BL biomarker levels and FU values were analysed and both absolute changes (follow-up minus baseline) and relative changes (absolute change divided by BL value, expressed as a percentage) were calculated. Associations between biomarkers and binary outcomes (e.g., DCB) were investigated using Wilcoxon rank-sum tests and logistic regression models. Discriminative performance was assessed with receiver operating characteristic (ROC) analysis and corresponding areas under the curve (AUC) with 95% confidence intervals. Missing data were not imputed, and analyses were performed using complete-case evaluation for all variables included in each model. Consequently, the effective sample size varied between analyses depending on data completeness. All eligible patients available during the study period were included. Statistical significance was defined as a two-sided p-value <0.05. All analyses were conducted in the R environment (version 4.4.0, R Foundation for Statistical Computing, Vienna, Austria).

## Results

### Patients’ characteristics and treatment response

A total of 179 patients treated in a palliative setting for advanced NSCLC with available imaging between January 2019 and December 2024 were screened. After excluding patients with active secondary malignancies (*n* = 13) and those included during ongoing systemic treatment (*n* = 1), 165 patients remained eligible for radiologic response assessment. Among them, 2 (1.2%) achieved complete remission, 44 (26.7%) partial remission, 82 (49.7%) SD and 37 (22.4%) progressive disease at first FU. Two patients with SD were lost to follow-up before 6 months and therefore excluded, resulting in a final study cohort of 80 patients with (i)SD, of whom 41 (51.3%) demonstrated DCB (for CONSORT diagram see Supplementary Fig. 1). All subsequent analyses were exclusively performed in this SD cohort.

Among the SD cohort, 40/80 patients (50%) were female with a median age of 65 years (range 36-85, IQR 58-75). Histology was predominantly adenocarcinoma (66/80, 83%), followed by squamous cell carcinoma (11/80, 14%). Regarding systemic antineoplastic therapy, chemo-immunotherapy was used in 39 patients (49%), immunotherapy alone in 27 (34%) and chemotherapy in 13 (16%) - one patient received chemotherapy plus targeted therapy. Molecular alterations included EGFR mutations in 16 patients (23%), ALK rearrangements in 3 (4.1%), BRAF mutations in 3 (4.6%) and a ROS1 fusion in one patient. PD-L1 positivity (TPS ≥ 1%) was observed in 60 of 79 evaluable patients (76%), with a median TPS of 20% (IQR 5-80). The availability of clinical and molecular variables varied across parameters, and sample sizes for regression analyses therefore differed according to data completeness. For detailed patient characteristics see Table 1.

**Table 1:** Patient characteristics of patients with stable disease at first restaging. ADC = Adenocarcinoma; ADC-SCC = Adeno-squamous carcinoma; CHT = Chemotherapy; CHT + ICI = Combined chemotherapy and immune checkpoint inhibitor; ICI = Immune checkpoint inhibitor; SCC = Squamous cell carcinoma.

Patient characteristics	n = 80^1^
Age	65 (58, 75)
Sex	
Male	40 (50%)
Female	40 (50%)
Metastasized	76 (95%)
Treatment naive	61 (76%)
Previous treatment lines	
None	61 (76%)
One	11 (14%)
More than 2	8 (10%)
Histology	
ADC	66 (83%)
SCC	11 (14%)
ADC-SCC	1 (1.3%)
Mucinous	1 (1.3%)
Sarcoid	1 (1.3%)
Regimen	
CHT + ICI	39 (49%)
ICI	27 (34%)
CHT	13 (16%)
ADC	1 (1.3%)
Smoking status	
Ever-smoker	73 (91%)
Never-smoker	5 (6.3%)
Not available	2 (2.5%)
Packyears (in ever-smoker)	40 (30, 60)
(missing)	10
EGFR mutation	16 (23%)
(missing)	10
ALK mutation	3 (4.1%)
(missing)	6
BRAF mutation	3 (4.6%)
(missing)	15
ROS1 mutation	1 (1.4%)
(missing)	7
PDL-1 positivity (TPS ≥ 1)	60 (76%)
(missing)	1
TPS [%]	20 (5, 80)
(missing)	20
Autoimmune disease	6 (7.9%)
(missing)	4
Cardiovascular disease	41 (53%)
(missing)	3
Pulmonary disease	19 (25%)
(missing)	3
Rheumatological disease	2 (2.6%)
(missing)	4
Inflammatory bowel disease	1 (1.3%)
(missing)	4
ECOG	
0	50 (63%)
1	27 (34%)
2	3 (3.8%)
Durable clinical benefit (DCB)	41 (51.3%)

1 Median (IQR); n (%)

**Fig. 1:**
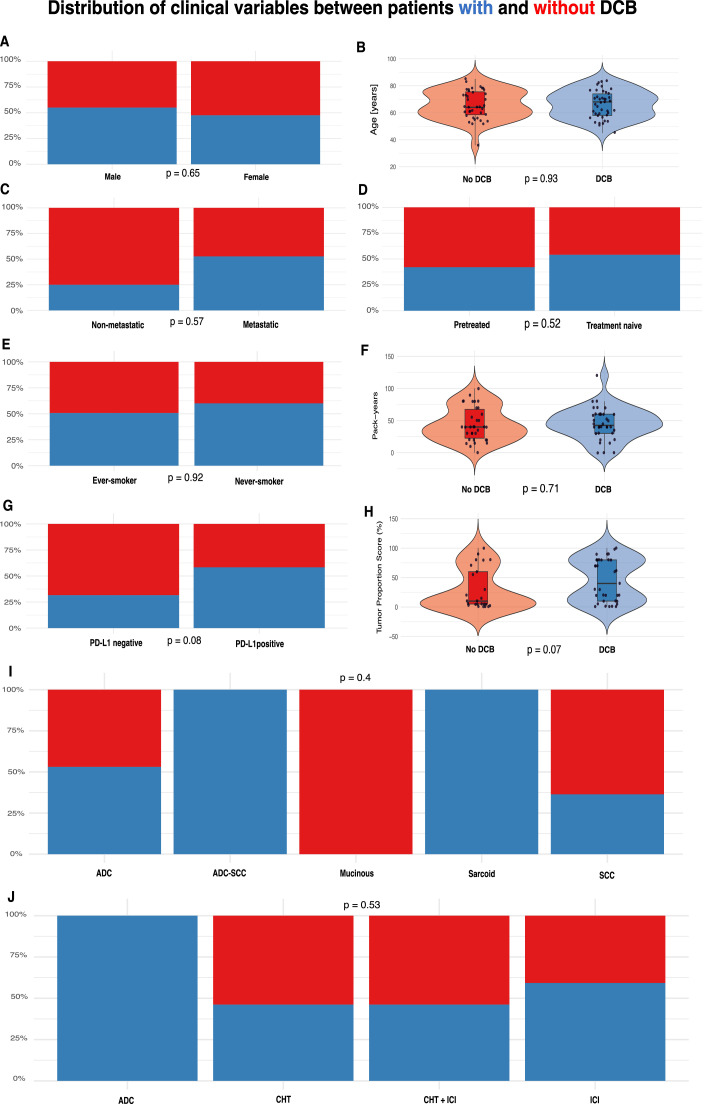
Distribution of clinical variables between patients with and without durable clinical benefit (DCB). (A) Sex; (B) Age; (C) Metastatic disease; (D) Treatment-naive status; (E) Smoking status; (F) Pack-years among ever-smokers; (G) PD-L1 positivity (TPS ≥ 1%); (H) Tumor proportion score (TPS); (I) Histology; (J) First-line treatment regimen. ADC = adenocarcinoma (in histology) and antibody-drug conjugate (in regimen); ADC-SCC = adenosquamous carcinoma; CHT = chemotherapy; CHT + ICI = combined chemotherapy and immune checkpoint inhibitor treatment; DCB = durable clinical benefit; ICI = immune checkpoint inhibitor; PD-L1 = programmed death-ligand 1; SCC = squamous cell carcinoma; TPS = tumor proportion score.

Patients with and without DCB showed overall comparable patient characteristics with regards to (media 68 years vs 64 years, *p* > 0.9), sex distribution (female 46% vs 54%, *p* = 0.7), rates of metastatic disease (98% vs 92%, *p* = 0.4), treatment-naivety (80% vs 72%, *p* = 0.4), smoking (ever-smoker 90% vs 92%, *p* > 0.9) and cumulative smoking exposure (43 vs 40 pack-years, *p* = 0.7). Also, histologic distribution did not differ between DCB and no-DCB groups (*p* = 0.3), as well as treatment regimens (*p* = 0.6), molecular alterations (all *p* ≥ 0.8), comorbidity patterns (all *p* ≥ 0.2), ECOG performance status (*p* = 0.061). PD-L1 positivity (TPS ≥ 1%) tended to be more frequent in the DCB group (85% vs 66%, *p* = 0.064) and tumor proportion score was higher (median 40% vs 10%, *p* = 0.072) - although neither reached statistical significance. These distributions are visualized in Fig. 1 and numerically shown in Table 2.

**Table 2:** Patient characteristics and durable clinical benefit (DCB). ADC = Adenocarcinoma; ADC-SCC = Adeno-squamous carcinoma; CHT = Chemotherapy; CHT + ICI = Combined chemotherapy and immune checkpoint inhibitor; ICI = Immune checkpoint inhibitor; SCC = Squamous cell carcinoma.

Patient characteristic	No DCB n = 39^1^	DCB n = 41^1^	p-value^2^
Age	64 (59, 76)	68 (58, 74)	>0.9
Sex			0.7
Male	18 (46%)	22 (54%)	
Female	21 (54%)	19 (46%)	
Metastasized	36 (92%)	40 (98%)	0.4
Treatment naive	28 (72%)	33 (80%)	0.4
Previous treatment lines			0.6
None	28 (72%)	33 (80%)	
One	7 (18%)	4 (9.8%)	
Two or more	4 (10.3%)	4 (9.7%)	
Histology			0.3
ADC	31 (79%)	35 (85%)	
SCC	7 (18%)	4 (9.8%)	
ADC-SCC	-	1 (2.4%)	
Mucinous	1 (2.6%)	-	
Sarcoid	-	1 (2.4%)	
Regimen			0.6
CHT + ICI	21 (54%)	18 (44%)	
ICI	11 (28%)	16 (39%)	
CHT	7 (18%)	6 (15%)	
ADC	-	1 (2.4%)	
Smoking status			>0.9
Ever-smoker	36 (92%)	37 (90%)	
Never-smoker	2 (5.1%)	3 (7.3%)	
Not available	1 (2.6%)	1 (2.4%)	
Packyears	40 (23, 68)	43 (30, 60)	0.7
(missing)	5	5	
EGFR mutation	9 (26%)	7 (20%)	0.8
(missing)	4	6	
ALK mutation	1 (2.9%)	2 (5.1%)	>0.9
(missing)	4	2	
BRAF mutation	1 (3.2%)	2 (5.9%)	>0.9
(missing)	8	7	
ROS1 mutation	-	1 (2.6%)	>0.9
(missing)	4	3	
PDL-1 positive (TPS ≥ 1)	25 (66%)	35 (85%)	0.064
(missing)	1	-	
TPS [%]	10 (5, 60)	40 (10, 80)	0.072
(missing)	14	6	
Autoimmune disease	4 (11%)	2 (5.1%)	0.4
(missing)	2	2	
Cardiovascular disease	22 (58%)	19 (49%)	0.5
(missing)	1	2	
Pulmonary disease	12 (32%)	7 (18%)	0.2
(missing)	1	2	
COPD	10 (26%)	6 (15%)	0.3
(missing)	1	2	
Rheumatological disease	2 (5.4%)	0 (0%)	0.2
(missing)	2	2	
Inflammatory bowel disease	1 (2.7%)	0 (0%)	0.5
(missing)	2	2	
ECOG			0.061
0	22 (56%)	28 (68%)	
1	17 (44%)	10 (24%)	
2	-	3 (7.3%)	

1 Median (IQR); n (%)

2 Wilcoxon rank sum test; Fisher's exact test; Wilcoxon rank sum exact test

To systematically evaluate clinical predictors of DCB, univariable logistic regression analyses were performed including age, sex, disease stage, treatment characteristics, molecular alterations, PD-L1 status, comorbidities, smoking history and performance status at treatment initiation. The only variable with a statistically significant effect was PD-L1 positivity, which was associated with increased odds of achieving DCB (OR 3.03, p = 0.047). All other clinical markers showed no significant association with DCB in univariable logistic regression analysis (Supplementary Table 1).

PD-L1 positivity, pulmonary disease, metastatic status and treatment-naivety were subsequently analysed as a multivariable logistic regression model. In this adjusted analysis, PD-L1 positivity remained the only independent predictor of DCB (aOR 3.30, *p* = 0.032). Pulmonary disease, metastatic status and treatment-naivety did not demonstrate significant contributions to the model (all *p* > 0.27, Supplementary Table 2).

### Inflammatory parameters and durable clinical benefit

Next, we analysed inflammatory blood-based biomarkers in the respective cohort as well as their longitudinal changes. Median time between BL blood draw at treatment initiation and at first FU was 63 days (range 22-125). Values at BL, FU, absolute and relative changes of named inflammatory parameter are depicted in Supplementary Table 3.

**Fig. 2:**
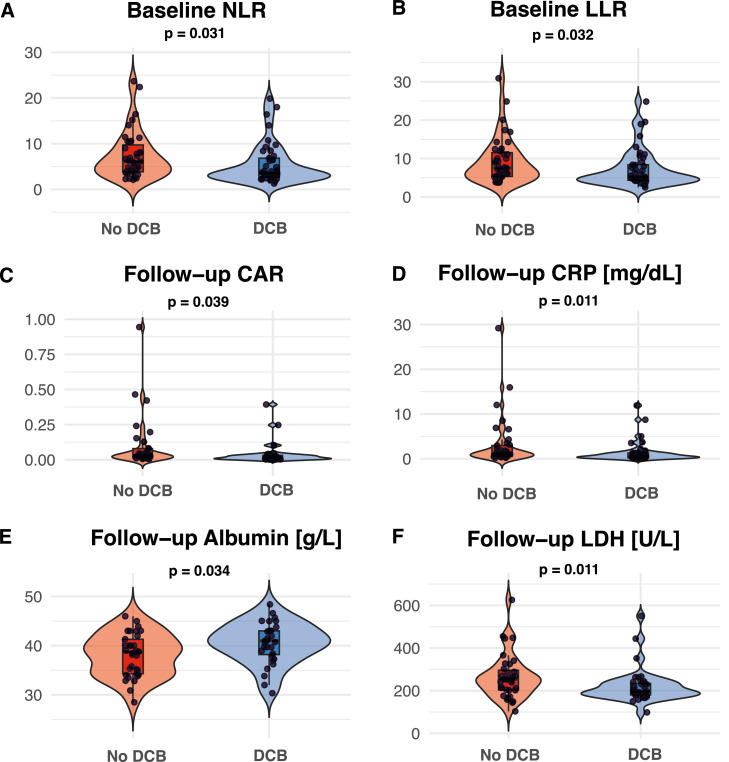
Distribution of inflammatory markers according to durable clinical benefit (DCB). (A) Baseline neutrophil-to-lymphocyte ratio (NLR); (B) Baseline lymphocyte-to-leukocyte ratio (LLR); (C) Follow-up C-reactive protein/albumin ratio (CAR); (D) Follow-up C-reactive protein (CRP); (E) Follow-up albumin; (F) Follow-up lactate dehydrogenase (LDH).

When comparing patients with and without DCB only several inflammatory markers differed between groups: At BL, patients with DCB had significantly lower NLR (3.6 vs 6.1, *p* = 0.031) and lower LLR (5.2 vs 7.5, *p* = 0.032). At first restaging, CRP (0.56 vs 1.18 mg/dL, *p* = 0.011), albumin (41.0 vs 38.7 g/L, *p* = 0.034) and CAR (0.02 vs 0.03, *p* = 0.039) were significantly lower in patients with DCB (Fig. 2). Other markers at BL or FU, as well as the absolute and relative changes across the treatment interval did not differ significantly (all *p* > 0.05, see Supplementary Table 4).

In univariable logistic regression only albumin at FU was associated with increased odds of achieving DCB (OR 1.15 per g/L, 95% CI 1.02-1.31, *p* = 0.032), whereas LDH at FU (*p* = 0.059), PLR (*p* = 0.071) and MLR at BL (*p* = 0.09) did not reach level of significance. The multivariable inflammatory model including follow-up albumin, follow-up LDH, baseline PLR and baseline MLR did not identify any single parameter as independently predictive of DCB (all *p* > 0.05). Results of the univariable and the multivariable logistic regression analysis are depicted in Supplementary Table 5 and 6, respectively.

### Comparison of clinical, inflammatory, and combined models for predicting DCB

To explore whether combining inflammatory biomarkers with clinical features may improve discrimination of DCB, we constructed an exploratory mixed multivariable logistic regression model integrating follow-up albumin, follow-up LDH, PD-L1 positivity and pulmonary comorbidity. In this combined model, higher follow-up albumin remained significantly associated with increased odds of achieving DCB (OR 1.16 per g/L, 95% CI 1.02-1.35, *p* = 0.022). PD-L1 positivity also demonstrated an independent association with higher likelihood of DCB (OR 4.07, 95% CI 1.14-17.12, p = 0.030). Follow-up LDH (*p* = 0.378) and pulmonary disease did not show a significant effect (*p* = 0.282, Supplementary Table 7).

**Fig. 3:**
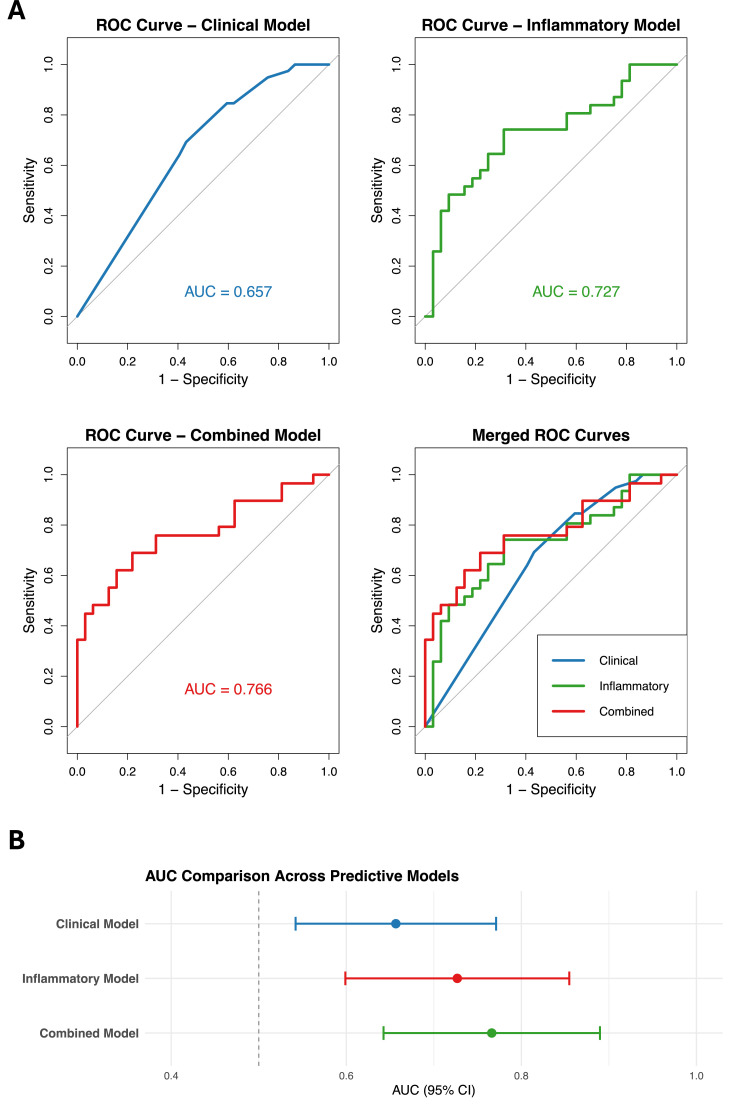
Discriminative performance of clinical, inflammatory, and combined models for durable clinical benefit (DCB). (A) Receiver operating characteristic (ROC) curves for the clinical model (PD-L1 positivity, pulmonary disease, metastatic status, treatment-naive status), the inflammatory model (follow-up albumin, follow-up LDH, baseline PLR, baseline MLR) and the combined model including both clinical and inflammatory predictors. (B) Comparison of area under the curve (AUC) values with 95% confidence intervals for the three models. The combined model shows numerically higher AUC, although differences are modest and should be interpreted cautiously given the exploratory design and limited sample size.

To quantify the discriminatory capacity of different modelling approaches, we generated ROC curves for the clinical, inflammatory and combined models. The clinical model, consisting of PD-L1 status, pulmonary comorbidity, metastatic status, and treatment-naivety, yielded an AUC of 0.657. The inflammatory model, incorporating follow-up albumin, follow-up LDH, baseline PLR, and baseline MLR, demonstrated improved discrimination with an AUC of 0.727. Ultimately, the combined model showed the highest apparent discriminative performance, achieving an AUC of 0.766 (Fig. 3), although the incremental improvement compared to the individual models was modest. Given the limited sample size and number of events, these model performance estimates should be interpreted cautiously.

## Discussion

In this exploratory biomarker study of a real-world cohort of patients with advanced NSCLC treated with systemic therapies, we investigated whether clinical characteristics and inflammatory blood markers may support early risk stratification among patients presenting with radiologic SD at first restaging. Our results demonstrate that while baseline clinical parameters alone show limited ability to discriminate DCB, selected inflammatory markers provide additional information. Importantly, the combination of clinical and inflammatory parameters showed the highest apparent discriminative performance for identifying patients who subsequently derive DCB after presenting with SD at the first re-staging.

The heterogeneity of patients classified as having SD after initial treatment represents a major clinical challenge. Radiologic SD may reflect true disease control, delayed treatment response or early resistance that precedes progression [12]. While radiologic response was assessed using standardized RECIST/iRECIST criteria, quantitative imaging features were not incorporated as such data were not consistently available in a standardized and analysis-ready format across the full real-world cohort. The present analysis therefore focused on routinely available clinical and laboratory parameters within this clinically defined subgroup. In our cohort, approximately half of all patients with SD at first restaging ultimately achieved DCB, underscoring the biological diversity within this response category and the need for refined risk stratification beyond imaging alone. By restricting all subsequent analyses to this clinically defined but biologically heterogeneous SD subgroup, we aimed to specifically address this unmet clinical question.

In our study, baseline clinical characteristics were largely balanced between patients with and without DCB. Although proportion of PD-L1 positivity was higher in patients with DCB, most demographic, clinical and treatment-related variables did not differ significantly between groups. Consistent with this, univariable and multivariable clinical models demonstrated only modest discriminative capacity by including only baseline clinical parameters. PD-L1 expression remained associated with DCB after adjustment in multivariable analysis, reinforcing its established role as a biomarker from immunotherapy, but also highlighting its limited standalone utility for early post-baseline decision-making within the SD population. Although ICI were not uniformly administered across the cohort, their predominant use may have contributed to the strength of the observed effect.

In contrast, several inflammatory parameters were associated with DCB in patients with SD at first restaging: At baseline, patients who later achieved DCB showed lower NLR and LLR. A low NLR has repeatedly been shown to be associated with improved survival and treatment response in several tumor entities, including NSCLC across disease stages and treatment modalities, including chemotherapy, radiotherapy and immunotherapy and is thought to reflect a balance toward antitumor lymphocyte-mediated immunity rather than neutrophil-driven systemic inflammation [13,14]. More recent data further support NLR as a surrogate of tumor immune status, correlating with tumor-infiltrating lymphocytes and immunotherapy efficacy [15,16]. Similarly, ratios incorporating lymphocyte proportions have been reported to carry prognostic information in lung cancer, underscoring the relevance of relative lymphocyte abundance as an indicator of effective antitumor immune surveillance [14]. Although the LLR itself is not a routinely used biomarker in clinical practice, it conceptually overlaps with established lymphocyte-based ratios such as the relative lymphocyte percentage and related composite ratios, all of which have consistently been associated with improved treatment response and survival outcomes in NSCLC [17,18]. Our findings align with this body of evidence and suggest that baseline immune cell balance contributes to subsequent DCB among patients with radiologic SD.

Additionally, at first restaging, patients who achieved DCB demonstrated lower CRP levels, higher serum albumin concentrations and a reduced CRP/albumin ratio. These parameters reflect early on-treatment biomarker states rather than purely baseline prognostic information. This observation is consistent with established inflammation-based prognostic frameworks such as the modified Glasgow Prognostic Score, which integrates CRP and albumin and has shown strong prognostic performance in NSCLC [19]. Notably, hypoalbuminemia has been shown to be associated with worse prognosis even in the absence of elevated CRP, underscoring the independent biological relevance of the nutritional status. Beyond lung cancer-specific cohorts, large prospective observational data across advanced malignancies further support albumin as a robust, independent prognostic marker, with low serum albumin levels being consistently associated with inferior survival, higher short-term mortality and impaired quality of life, particularly in patients with cancer cachexia [20]. Furthermore, the association between FU LDH and reduced odds of DCB in our cohort aligns with prior work defining LDH as a marker of tumor burden and metabolic activity [21].

Interestingly, previous studies showed that combined inflammatory and nutritional biomarkers in advanced NSCLC treated with ICI outperform TNM staging for risk prediction and can be integrated alongside clinical variables such as age, sex, smoking history and histopathological type [22]. For early-stage disease, risk models incorporating radiomics features from CT imaging, tumor size and patient age have demonstrated superior prognostic accuracy compared to clinicopathological data alone [23,24]. In our study, the apparent discriminative performance of laboratory parameters was numerically higher when combined with selected clinical parameters. The mixed clinical-inflammatory model incorporating FU albumin, FU LDH, PD-L1 status and pulmonary comorbidity showed the highest AUC, although this difference was modest and should be interpreted cautiously given the limited sample size and potential for model optimism. However, this finding is biologically plausible, as chronic pulmonary diseases represent the most prevalent comorbidities in NSCLC and have been consistently associated with inferior overall and cancer-specific survival, independent of tumor stage. Population-based analyses have demonstrated that respiratory comorbidities contribute significantly to higher mortality in lung cancer, likely through impaired functional reserve, chronic systemic inflammation and limitations in treatment delivery [25,26]. Taken together, these data emphasize that inflammatory biomarkers are most informative when interpreted within the appropriate clinical context.

Our study adds to emerging evidence that “disease stability” is not a biologically uniform state. In the immunotherapy era, delayed responses or pseudo-progression may occur, complicating early response assessment [27]. The observation that the combined model showed numerically higher discriminative performance compared to biomarker- and clinical-only models suggests that integrating clinical and inflammatory parameters may support early risk stratification, although the improvement is modest and requires prospective validation. Future studies should thus focus on composite models combining clinical, molecular and imaging-derived features to refine post-baseline prediction, especially among patients with SD.

However, this study has several limitations that warrant consideration. First, its retrospective design, the relatively small sample size and the limited number of outcome events restrict statistical power, limit generalizability and may increase the risk of model instability and overfitting without clear evidence of meaningful improvement in discrimination in multivariable analyses. Given the number of events relative to the number of candidate predictors, the regression models, particularly the combined clinical-inflammatory model, should be interpreted as exploratory and hypothesis-generating. Accordingly, the apparent discriminative performance of the models may be optimistic and requires validation in larger independent cohorts. Missing data for key clinical variables, including PD-L1 tumor proportion score and molecular alterations, further restricted the ability to fully adjust for all clinically relevant covariates. As analyses were performed using complete-case evaluation, this may have introduced selection bias if patients with available biomarker data differed systematically from those with missing data. Second, the definition of DCB inherently relies on remaining progression-free and alive for ≥ 6 months, introducing a time-dependent component. Patients experiencing early progression or death are classified as non-DCB by design, which is clinically reasonable but may introduce bias toward patients with more favorable early disease courses. Accordingly, DCB should be interpreted as an endpoint for discrimination within the stable disease subgroup rather than as a direct surrogate for long-term survival outcomes. Third, analyses incorporating follow-up biomarkers and biomarker dynamics are vulnerable to time-related biases, including guarantee-time bias. Follow-up biomarkers were assessed only in patients who remained clinically stable and evaluable until first restaging, which may preferentially select patients with more favorable early disease courses. In addition, the interval between baseline and first follow-up varied substantially across patients, and the timing of blood sampling relative to imaging was not fully standardized. These factors may influence the interpretation of follow-up biomarker levels and their changes over time. Accordingly, follow-up parameters should be understood as early on-treatment biomarkers rather than unbiased baseline prognostic markers, and their association with durable clinical benefit should be interpreted with caution. Fourth, biomarkers were measured only at BL and first FU - more longitudinal sampling could have better captured dynamic trajectories and their relation to treatment efficacy. Another limitation is that imaging data were used exclusively for response categorization according to RECIST or iRECIST criteria, without incorporation of additional quantitative imaging features. Including additional radiological variables such as tumor burden, number of (non-) target lesions, affected organ system into multivariable models in a small, clinically selected subgroup in the context of a limited number of events may increase the risk of model instability and overfitting without clearly improving discrimination. The present study was designed to evaluate routinely available clinical and laboratory parameters, hence future studies in larger cohorts with pre-defined imaging pipelines should aim to assess whether integrating tumor burden metrics, lesion distribution, or radiomics features provides additional value beyond clinical and inflammatory markers in this setting. Finally, our study population was treated heterogeneously, including chemotherapy, immunotherapy and combined regimens. Residual confounders by treatment choice, prior therapies, or unmeasured factors cannot be excluded. Therefore, our results should be interpreted as hypothesis-generating and warrant prospective validation in larger, more homogeneous cohorts with standardized biomarker assessment intervals.

Despite these limitations, our study also has several strengths. First, it represents a well-characterized, real-world cohort of advanced NSCLC patients treated in routine clinical practice, thereby reflecting the heterogeneity encountered outside of clinical trials. The inclusion of diverse histologies, treatment regimens and PD-L1 expression levels increases the external validity of our findings. Inflammatory markers were measured prospectively at predefined time points (BL and first FU). This minimizes misclassification and ensures that biomarker changes correspond to clinically meaningful restaging timepoints. Furthermore, we systematically assessed biomarker performance using multiple complementary approaches - univariable, multivariable, as well as ROC curve analysis, providing a robust evaluation of both discrimination and prognostic relevance. Additionally, the study specifically focused on the clinically relevant subgroup of patients with SD at first FU, a population in whom treatment decision-making is particularly challenging. By combining clinical covariates with inflammatory marker data, we were able to demonstrate meaningful discrimination of DCB within this otherwise understudied subgroup.

In this real-world cohort of patients with advanced NSCLC and radiologic SD at first restaging, inflammatory biomarkers provided clinically relevant information for identifying patients who derive DCB from systemic therapy. Lower systemic inflammation, reflected by reduced inflammatory ratios at baseline and higher albumin levels at early follow-up, was associated with a higher likelihood of DCB. While baseline clinical characteristics alone showed restricted predictive capacity, the integration of selected inflammatory parameters with established clinical factors, particularly PD-L1 expression, yielded the highest predictive performance. These hypothesis-generating findings suggest that routinely available inflammatory blood markers may complement clinical assessment for early risk stratification within the heterogeneous SD population. Prospective validation in larger, independent cohorts is warranted to confirm their role in clinical decision-making.

## Fundings

The financial support by the Austrian Federal Ministry for Digital and Economic Affairs, the National Foundation for Research, Technology and Development and the Christian Doppler Research Association is gratefully acknowledged.

The funders had no influence on the study’s conception, methodology, data handling, results or conclusions.

All named authors meet all four criteria for (co)-authorship provided by the Good Scientific Practice guidelines of the Medical University of Vienna. All authors read and approved the final manuscript.

## Declarations

### Ethics approval and consent to participate

This single-centred, retrospective real-world observational cohort study was approved by the Ethics Committee of the *Medical University of Vienna* (ethic committee number 2065/2022) in accordance with the Declaration of Helsinki.

### Consent for publication

Not applicable.

### Availability of data and material

Further data will be shared upon reasonable request to the corresponding author.

### Statement on the use of artificial intelligence

Artificial intelligence (AI) tools were used to support specific aspects of this study. ChatGPT (OpenAI, GPT-5) was employed to assist with R programming for statistical analyses, data visualization and Fig. generation, as well as to refine the clarity and scientific tone of the manuscript text. All conceptualization, study design, data interpretation and critical reasoning were conducted by the authors. The AI tools served only as supportive instruments for efficiency and language refinement. All AI-generated content was rigorously reviewed, validated, and edited by the authors, who accept full responsibility for the integrity and accuracy of the final manuscript.

## CRediT authorship contribution statement

**Kleinberger M:** Writing – original draft, Visualization, Project administration, Methodology, Investigation, Formal analysis, Data curation, Conceptualization. **Laengle S:** Writing – review & editing, Data curation. **Berger JM:** Writing – review & editing, Data curation. **Gottmann L:** Writing – review & editing, Data curation. **Sunder-Plassmann V:** Writing – review & editing, Data curation. **Korpan M:** Writing – review & editing, Data curation. **Solano Henao I:** Writing – review & editing, Data curation. **Fuerst J:** Writing – review & editing, Data curation. **Starzer AM:** Writing – review & editing, Data curation. **Berchtold L:** Writing – review & editing, Investigation, Formal analysis. **Tomasich E:** Writing – review & editing, Methodology, Investigation. **Preusser M:** Writing – review & editing, Supervision, Resources, Funding acquisition. **Furtner J:** Writing – review & editing, Supervision, Methodology, Investigation, Formal analysis, Data curation. **Berghoff AS:** Writing – review & editing, Supervision, Resources, Project administration, Investigation, Funding acquisition, Conceptualization.

## Declaration of competing interest

M.K. received travel support from Eli Lilly Pharmaceutical via institutional nomination.

J.M.B. received travel support from Amgen via institutional nomination and honoraria for lectures, consultation or advisory board participation from MSD.

A.M.S. has received honoraria for lectures from AstraZeneca and travel and congress registration support from PharmaMar, MSD, Lilly, AstraZeneca and Stemline Menarini.

A.S.B. received research support from Daiichi Sankyo, Roche, and honoraria for lectures, consultation or advisory board participation from Roche, Bristol-Meyers Squibb, Merck, Daiichi Sankyo as well as travel support from Roche, Amgen and AbbVie.

M.P. has received honoraria for lectures, consultation or advisory board participation from the following for-profit companies: Bayer, Bristol-Myers Squibb, Novartis, Gerson Lehrman Group (GLG), CMC Contrast, GlaxoSmithKline, Mundipharma, Roche, BMJ Journals, MedMedia, Astra Zeneca, AbbVie, Lilly, Medahead, Daiichi Sankyo, Sanofi, Merck Sharp & Dome, Tocagen, Adastra, Gan & Lee Pharmaceuticals, Janssen, Servier, Miltenyi, Böhringer-Ingelheim, Telix, Medscape, OncLive, Medac, Nerviano Medical Sciences, ITM Oncologics GmbH, AdAcAp.

All other co-authors have no conflict of interest to report. All other co-authors have no conflict of interest to report.

## Figures and Tables

**Table:** 

Term	Definition
ICI	Immune Checkpoint Inhibitor; immunotherapy targeting PD-1/PD-L1 or CTLA-4 pathways to enhance antitumor immune responses.
RECIST	Response Evaluation Criteria in Solid Tumors; standard radiologic criteria for assessing tumor response based on measurable lesion size.
iRECIST	Immune RECIST; modified guidelines for immunotherapy incorporating atypical patterns such as pseudo progression.
DCB	Durable Clinical Benefit; sustained disease control (CR/PR/SD) lasting ≥ 6 months.
CR	Complete Response; disappearance of all measurable lesions.
PR	Partial Response; ≥ 30% decrease in the sum of target lesion diameters and no new tumor lesions.
SD	Stable Disease; insufficient shrinkage for PR (< 30% decrease) and insufficient increase for PD (< 20% increase) and no new tumor lesions.
PD	Progressive Disease; ≥ 20% increase in lesion size or appearance of new lesions.

**Table:** 

ALK	Anaplastic Lymphoma Kinase
AUC	Area Under the Curve
BRAF	B-Raf Proto-Oncogene
BL	Baseline
CI	Confidence Interval
CR	Complete Response
CRP	C-Reactive Protein
DCB	Durable Clinical Benefit
ECOG	Eastern Cooperative Oncology Group
EGFR	Epidermal Growth Factor Receptor
FU	Follow-Up
ICI	Immune Checkpoint Inhibitor
iRECIST	Immune Response Evaluation Criteria in Solid Tumors
MSI	Microsatellite Instability
NSCLC	Non-Small Cell Lung Cancer
OR	Odds Ratio
PACS	Picture Archiving and Communication System
PD	Progressive Disease
PD-L1	Programmed Death-Ligand 1
PR	Partial Response
RECIST	Response Evaluation Criteria in Solid Tumors
ROS1	c-ros Oncogene 1
SD	Stable Disease
TMB	Tumor Mutational Burden
TPS	Tumor Proportion Score

## Appendix B Supplementary materials

Image, application 1
