# Supplementary material for: Inflammatory biomarkers refine progression risk stratification in NSCLC patients with stable disease
Source: Transl Oncol. 2026 Apr 20;68:102769. doi: 10.1016/j.tranon.2026.102769 (PMC13123324; doi:10.1016/j.tranon.2026.102769)
Supplement: Supplementary file 1 [file mmc1.docx]

# **Supplementary Data**

# **Supplementary Tables**

| **Variable** | **OR** | **95% CI** | **p-value** |
| --- | --- | --- | --- |
| **Age** | 1.00 | (0.96, 1.05) | 0.914 |
| **Sex** | 0.74 | (0.30, 1.78) | 0.503 |
| **Metastasized** | 3.33 | (0.41, 69.04) | 0.307 |
| **Treatment naive** | 1.62 | (0.58, 4.72) | 0.363 |
| **Previous treatment lines**^x^ | - | - | 0.411 |
| **Histology**^x^ | - | - | 0.269 |
| **Regimen**^x^ | - | - | 0.462 |
| **Smoking status** | 0.69 | (0.09, 4.37) | 0.688 |
| **Pack years** | 1.00 | (0.98, 1.02) | 0.917 |
| **EGFR mutation** | 0.72 | (0.23, 2.21) | 0.570 |
| **ALK mutation** | 1.84 | (0.17, 40.60) | 0.626 |
| **BRAF mutation** | 1.87 | (0.17, 41.58) | 0.615 |
| **ROS1 mutation**^o^ | - | - | 0.992 |
| **PDL-1 positivity** | 3.03 | (1.05, 9.66) | **0.047** |
| **TPS [%]** | 1.01 | (1.00, 1.03) | 0.086 |
| **Autoimmune disease** | 0.45 | (0.06, 2.44) | 0.369 |
| **Cardiovascular disease** | 0.69 | (0.28, 1.69) | 0.420 |
| **Pulmonary disease** | 0.47 | (0.16, 1.35) | 0.169 |
| **Rheumatological disease**^o^ | - | - | 0.992 |
| **Inflammatory bowel disease**^o^ | - | - | 0.991 |
| **ECOG** | 0.87 | (0.39, 1.89) | 0.717 |

**Supplementary Table 1. Univariable logistic regression analysis of clinical baseline characteristics associated with durable clinical benefit (DCB).** **^x^**Variables as multi-level categorical predictors for which no single odds ratio can be reported. **^o^**Variables as rare-event predictors where effect estimates could not be reliably computed. p-values reflect overall likelihood ratio tests.

| **Variable** | **OR** | **95% CI** | **p value** |
| --- | --- | --- | --- |
| **PD-L1 positivity** | 3.30 | (1.11, 11.09) | **0.032** |
| **Pulmonary disease** | 0.54 | (0.17, 1.64) | 0.278 |
| **Metastatic disease** | 1.86 | (0.20, 25.28) | 0.592 |
| **Treatment-naive** | 1.13 | (0.31, 4.23) | 0.847 |

**Supplementary Table 2. Multivariable analysis of clinical parameters and durable clinical benefit (DCB).**

| **Inflammatory parameter** | **n = 80**^1^ |
| --- | --- |
| **Baseline / Treatment initiation** |  |
| **WBC** [G/L] | 9.2 (6.9, 11.6) |
| **ANC** [G/L] | 6.78 (4.87, 8.25) |
| **ALC** [G/L] | 1.20 (0.87, 2.05) |
| **AMC** [G/L] | 0.70 (0.50, 0.90) |
| **PLT** [G/L] | 292 (234, 375) |
| **NLR** | 4.7 (3.1, 8.2) |
| **LLR** | 6.3 (4.5, 10.3) |
| **PLR** | 202 (158, 354) |
| **MLR** | 0.50 (0.35, 0.77) |
| **CRP/Albumin ratio** | 0.02 (0.01, 0.08) |
| **CRP** [mg/dL] | 1.0 (0.4, 3.3) |
| **Albumin** [g/L] | 40.3 (36.5, 44.2) |
| **LDH** [U/L] | 207 (175, 277) |
| **Follow-up / First restaging** |  |
| **WBC** [G/L] | 6.1 (4.8, 8.7) |
| **ANC** [G/L] | 3.58 (2.48, 5.80) |
| **ALC** [G/L] | 1.23 (0.90, 1.63) |
| **AMC** [G/L] | 0.72 (0.50, 0.90) |
| **PLT** [G/L] | 282 (216, 362) |
| **NLR** | 3.09 (1.82, 4.79) |
| **LLR** | 4.92 (3.40, 6.44) |
| **PLR** | 234 (154, 350) |
| **MLR** | 0.56 (0.35, 0.72) |
| **CRP/Albumin ratio** | 0.02 (0.01, 0.05) |
| **CRP** [mg/dL] | 0.91 (0.40, 1.80) |
| **Albumin** [g/L] | 39.8 (35.7, 43.0) |
| **LDH** [U/L] | 224 (184, 264) |
| **Absolute change** |  |
| **WBC** [G/L] | -2.0 (-4.9, -0.3) |
| **ANC** [G/L] | -2.1 (-4.5, -0.3) |
| **ALC** [G/L] | 0.00 (-0.40, 0.30) |
| **AMC** [G/L] | 0.06 (-0.24, 0.26) |
| **PLT** [G/L] | -3 (-71, 85) |
| **NLR** | -1.6 (-3.7, -0.3) |
| **LLR** | -1.7 (-3.9, -0.2) |
| **PLR** | -9 (-87, 79) |
| **MLR** | -0.01 (-0.26, 0.15) |
| **CRP/Albumin ratio** | 0.00 (-0.04, 0.02) |
| **CRP** [mg/dL] | 0.0 (-1.6, 0.8) |
| **Albumin** [g/L] | -1.0 (-4.5, 2.5) |
| **LDH** [U/L] | 19 (-47, 61) |
| **Relative change** [%] |  |
| **WBC** [G/L] | -25.0 (-52.7, -2.0) |
| **ANC** [G/L] | -42.9 (-60.4, -9.0) |
| **ALC** [G/L] | 0.1 (-24.3, 28.3) |
| **AMC** [G/L] | 10.0 (-37.1, 40.2) |
| **PLT** [G/L] | -0.6 (-25.2, 34.0) |
| **NLR** | -39.0 (-64.5, -5.9) |
| **LLR** | -30.3 (-48.2, -3.0) |
| **PLR** | -3.8 (-34.8, 48.3) |
| **MLR** | -4.4 (-33.2, 40.6) |
| **CRP/Albumin ratio** | 0.3 (-71.4, 180.4) |
| **CRP** [mg/dL] | -8.9 (-78.1, 154.9) |
| **Albumin** [g/L] | -2.5 (-10.4, 7.2) |
| **LDH** [U/L] | 11.9 (-17.1, 22.7) |
| ^1^Median (IQR); n (%) | |

**Supplementary Table 3. Inflammatory parameter in patients with stable diseases.** ALC = absolute lymphocyte count; AMC = absolute monocyte count; ANC = absolute neutrophil count; LLR = lymphocyte-to-leukocyte ratio; MLR = monocyte-to-lymphocyte ratio; NLR = neutrophil-to-lymphocyte ratio; PLR = platelet-to-lymphocyte ratio; PLT = platelet count; WBC = white blood cell count.

| **Patient characteristic** | **No DCB**  n = 39^1^ | **DCB**  n = 41^1^ | **p-value**^2^ |
| --- | --- | --- | --- |
| **Baseline / Treatment initiation** |  |  |  |
| **WBC** [G/L] | 9.2 (7.6, 11.4) | 9.2 (6.1, 12.9) | 0.5 |
| **ANC** [G/L] | 7.20 (5.60, 8.55) | 6.10 (4.20, 7.83) | 0.15 |
| **ALC** [G/L] | 1.13 (0.80, 1.77) | 1.30 (1.02, 2.13) | 0.2 |
| **AMC** [G/L] | 0.74 (0.53, 0.99) | 0.67 (0.46, 0.84) | 0.3 |
| **PLT** [G/L] | 298 (227, 390) | 278 (238, 354) | 0.5 |
| **NLR** | 6.1 (3.8, 9.7) | 3.6 (2.7, 6.8) | **0.031** |
| **LLR** | 7.5 (5.3, 11.5) | 5.2 (4.4, 8.4) | **0.032** |
| **PLR** | 239 (162, 411) | 192 (154, 280) | 0.2 |
| **MLR** | 0.56 (0.38, 1.06) | 0.46 (0.29, 0.67) | 0.12 |
| **CRP/Albumin ratio** | 0.03 (0.01, 0.10) | 0.02 (0.01, 0.08) | 0.3 |
| **CRP** [mg/dL] | 1.2 (0.4, 3.8) | 0.8 (0.5, 2.8) | 0.5 |
| **Albumin** [g/L] | 40.0 (36.6, 43.4) | 41.0 (36.6, 44.5) | 0.5 |
| **LDH** [U/L] | 218 (185, 278) | 196 (162, 264) | 0.4 |
| **Follow-up / First restaging** |  |  |  |
| **WBC** [G/L] | 5.9 (4.4, 9.1) | 6.2 (5.0, 8.0) | 0.8 |
| **ANC** [G/L] | 4.09 (2.20, 5.70) | 3.50 (2.92, 5.83) | 0.6 |
| **ALC** [G/L] | 1.20 (0.81, 1.44) | 1.30 (0.98, 1.75) | 0.3 |
| **AMC** [G/L] | 0.79 (0.50, 0.90) | 0.71 (0.50, 0.90) | >0.9 |
| **PLT** [G/L] | 282 (197, 394) | 283 (239, 349) | 0.7 |
| **NLR** | 3.13 (1.98, 4.32) | 3.00 (1.79, 4.91) | 0.8 |
| **LLR** | 5.00 (3.68, 6.31) | 4.77 (3.26, 7.09) | 0.7 |
| **PLR** | 258 (155, 410) | 226 (154, 325) | 0.4 |
| **MLR** | 0.64 (0.36, 0.85) | 0.48 (0.35, 0.67) | 0.3 |
| **CRP/Albumin ratio** | 0.03 (0.01, 0.08) | 0.02 (0.01, 0.03) | **0.039** |
| **CRP** [mg/dL] | 1.18 (0.59, 2.95) | 0.56 (0.22, 1.27) | **0.011** |
| **Albumin** [g/L] | 38.7 (34.3, 41.3) | 41.0 (38.2, 43.0) | **0.034** |
| **LDH** [U/L] | 253 (204, 296) | 196 (180, 234) | **0.011** |
| **Absolute change** |  |  |  |
| **WBC** [G/L] | -3.3 (-5.3, 0.1) | -1.4 (-4.3, -0.3) | 0.5 |
| **ANC** [G/L] | -3.0 (-5.2, -1.0) | -1.7 (-4.0, -0.1) | 0.3 |
| **ALC** [G/L] | -0.01 (-0.32, 0.19) | 0.07 (-0.41, 0.34) | 0.5 |
| **AMC** [G/L] | -0.04 (-0.29, 0.20) | 0.10 (-0.21, 0.30) | 0.3 |
| **PLT** [G/L] | -7 (-93, 76) | 6 (-34, 87) | 0.4 |
| **NLR** | -1.7 (-4.5, -0.3) | -1.3 (-3.0, -0.3) | 0.4 |
| **LLR** | -2.0 (-4.7, -0.2) | -1.2 (-3.1, -0.2) | 0.4 |
| **PLR** | -9 (-101, 63) | -2 (-78, 88) | 0.8 |
| **MLR** | -0.04 (-0.32, 0.13) | 0.00 (-0.20, 0.16) | 0.4 |
| **CRP/Albumin ratio** | 0.00 (-0.03, 0.02) | 0.00 (-0.03, 0.02) | 0.5 |
| **CRP** [mg/dL] | 0.1 (-1.4, 0.9) | -0.1 (-1.7, 0.7) | 0.4 |
| **Albumin** [g/L] | -1.2 (-4.7, 2.4) | 0.0 (-4.2, 2.4) | 0.5 |
| **LDH** [U/L] | 37 (-46, 70) | 3 (-45, 30) | 0.12 |
| **Relative change** [%] |  |  |  |
| **WBC** [G/L] | -35.2 (-54.4, 1.9) | -21.7 (-45.7, -4.2) | 0.3 |
| **ANC** [G/L] | -55.0 (-68.3, -15.3) | -40.8 (-57.4, -1.7) | 0.086 |
| **ALC** [G/L] | -2.5 (-17.9, 17.5) | 9.3 (-24.3, 35.3) | 0.7 |
| **AMC** [G/L] | -5.3 (-47.1, 35.8) | 15.5 (-31.3, 49.6) | 0.4 |
| **PLT** [G/L] | -2.7 (-29.8, 37.0) | 2.3 (-10.7, 30.7) | 0.3 |
| **NLR** | -41.3 (-68.1, -6.5) | -37.1 (-58.7, -6.9) | 0.4 |
| **LLR** | -31.2 (-53.7, -2.7) | -25.0 (-41.6, -5.6) | 0.5 |
| **PLR** | -3.8 (-33.8, 50.2) | -2.1 (-34.8, 44.1) | >0.9 |
| **MLR** | -6.6 (-36.5, 48.6) | -2.2 (-30.5, 38.6) | 0.7 |
| **CRP/Albumin ratio** | 15.6 (-53.7, 201.5) | -19.9 (-80.6, 152.1) | 0.3 |
| **CRP** [mg/dL] | 12.6 (-54.5, 177.8) | -41.2 (-81.0, 149.7) | 0.2 |
| **Albumin** [g/L] | -3.4 (-11.1, 6.4) | -1.0 (-9.2, 7.0) | 0.6 |
| **LDH** [U/L] | 19.5 (-17.5, 41.6) | 1.2 (-16.6, 18.1) | 0.2 |
| ^1^Median (IQR); n (%) | | | |
| ^2^Wilcoxon rank sum test; Fisher's exact test; Wilcoxon rank sum exact test | | | |

**Supplementary Table 4.** **Inflammatory parameter and durable clinical benefit (DCB).** ALC = absolute lymphocyte count; AMC = absolute monocyte count; ANC = absolute neutrophil count; LLR = lymphocyte-to-leukocyte ratio; MLR = monocyte-to-lymphocyte ratio; NLR = neutrophil-to-lymphocyte ratio; PLR = platelet-to-lymphocyte ratio; PLT = platelet count; WBC = white blood cell count.

| **Inflammatory parameter** | **OR** | **95% CI** | **p value** |
| --- | --- | --- | --- |
| **FU Albumin** | 1.15 | (1.02, 1.31) | **0.0316** |
| **FU LDH** | 0.99 | (0.99, 1.00) | 0.0589 |
| **BL PLR** | 1.00 | (1.00, 1.00) | 0.0712 |
| **BL MLR** | 0.40 | (0.12, 0.99) | 0.0900 |
| **FU CRP** | 0.87 | (0.71, 1.00) | 0.1067 |
| **BL LLR** | 0.93 | (0.85, 1.01) | 0.1078 |
| **BL NLR** | 0.92 | (0.83, 1.01) | 0.1150 |
| **Relative Change of LDH** | 0.99 | (0.98, 1.00) | 0.1226 |
| **FU CRP/Albumin ratio** | 0.02 | (0.00, 1.69) | 0.1732 |
| **BL ALC** | 1.42 | (0.87, 2.47) | 0.1769 |
| **FU ALC** | 1.45 | (0.84, 2.92) | 0.2371 |
| **Relative Change of ANC** | 1.01 | (1.00, 1.02) | 0.2371 |
| **Relative Change of Albumin** | 1.02 | (0.99, 1.06) | 0.2436 |
| **BL AMC** | 0.52 | (0.15, 1.67) | 0.2824 |
| **Relative Change of LLR** | 1.00 | (1.00, 1.01) | 0.3648 |
| **Relative Change of CRP/Albumin ratio** | 1.00 | (1.00, 1.00) | 0.4052 |
| **BL Albumin** | 1.04 | (0.95, 1.13) | 0.4147 |
| **FU PLR** | 1.00 | (1.00, 1.00) | 0.4218 |
| **Relative Change of WBC** | 1.01 | (0.99, 1.02) | 0.4336 |
| **Relative Change of CRP** | 1.00 | (1.00, 1.00) | 0.4677 |
| **FU PLT** | 1.00 | (1.00, 1.00) | 0.4881 |
| **BL PLT** | 1.00 | (1.00, 1.00) | 0.5021 |
| **FU AMC** | 1.48 | (0.46, 5.71) | 0.5232 |
| **Relative Change of MLR** | 1.00 | (1.00, 1.00) | 0.5371 |
| **BL ANC** | 0.96 | (0.83, 1.10) | 0.5485 |
| **Relative Change of NLR** | 1.00 | (1.00, 1.01) | 0.5578 |
| **FU MLR** | 1.25 | (0.62, 3.50) | 0.5674 |
| **FU NLR** | 0.96 | (0.82, 1.11) | 0.5703 |
| **FU LLR** | 0.97 | (0.84, 1.12) | 0.6828 |
| **BL WBC** | 0.98 | (0.87, 1.11) | 0.7599 |
| **Relative Change of PLT** | 1.00 | (0.99, 1.01) | 0.7666 |
| **Relative Change of PLR** | 1.00 | (1.00, 1.00) | 0.8116 |
| **BL LDH** | 1.00 | (1.00, 1.00) | 0.8225 |
| **Relative Change of AMC** | 1.00 | (1.00, 1.00) | 0.8426 |
| **Relative Change of ALC** | 1.00 | (0.99, 1.01) | 0.8614 |
| **FU WBC** | 0.99 | (0.86, 1.14) | 0.8917 |
| **BL CRP** | 0.99 | (0.89, 1.10) | 0.9046 |
| **BL CRP/Albumin ratio** | 0.84 | (0.03, 21.73) | 0.9170 |
| **FU ANC** | 1.00 | (0.84, 1.19) | 0.9944 |

**Supplementary Table 5. Univariable logistic regression analysis of baseline, follow-up and relative change of inflammatory parameter associated with durable clinical benefit (DCB).**

| **Inflammatory parameter** | **OR** | **95% CI** | **p value** |
| --- | --- | --- | --- |
| **Follow-up Albumin** | 1.08 | (0.95, 1.23) | 0.247 |
| **Follow-up LDH** | 1.00 | (0.99, 1.00) | 0.097 |
| **Baseline PLR** | 1.00 | (1.00, 1.00) | 0.550 |
| **Baseline MLR** | 0.57 | (0.12, 2.49) | 0.465 |

**Supplementary Table 6. Multivariable analysis of inflammatory parameters and durable clinical benefit (DCB).**

| **Combined parameter** | **OR** | **95% CI** | **p value** |
| --- | --- | --- | --- |
| **Follow-up Albumin** | 1.16 | (1.02, 1.35) | **0.022** |
| **Follow-up LDH** | 1.00 | (0.99, 1.00) | 0.378 |
| **PD-L1 positivity** | 4.07 | (1.14, 17.12) | **0.030** |
| **Pulmonary disease** | 0.50 | (0.13, 1.76) | 0.282 |

**Supplementary Table 7. Multivariable analysis of inflammatory and clinical parameters and durable clinical benefit (DCB).**

# **Supplementary Figures**


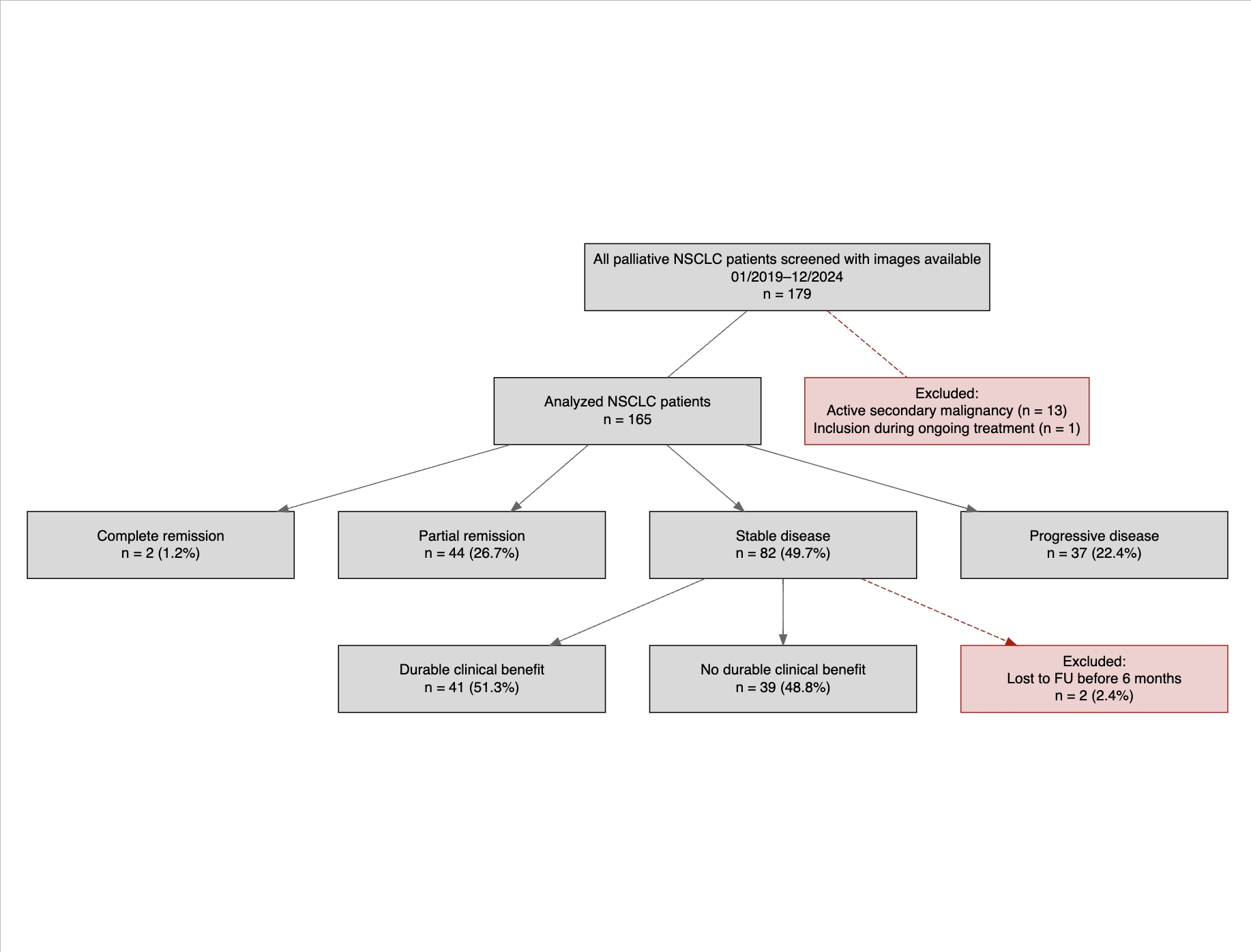


**Supplementary Figure 1. CONSORT diagram of enrolled NSCLC patients.**
